# Microfluidic devices: The application in TME modeling and the potential in immunotherapy optimization

**DOI:** 10.3389/fgene.2022.969723

**Published:** 2022-09-08

**Authors:** Yuting Li, Honghong Fan, Junli Ding, Junying Xu, Chaoying Liu, Huiyu Wang

**Affiliations:** Department of Oncology, Wuxi People’s Hospital Affiliated to Nanjing Medical University, Wuxi, China

**Keywords:** microfluidics, microfluidic devices, TME, tumor immunotherapy, multi-omic analyses

## Abstract

With continued advances in cancer research, the crucial role of the tumor microenvironment (TME) in regulating tumor progression and influencing immunotherapy outcomes has been realized over the years. A series of studies devoted to enhancing the response to immunotherapies through exploring efficient predictive biomarkers and new combination approaches. The microfluidic technology not only promoted the development of multi-omics analyses but also enabled the recapitulation of TME *in vitro* microfluidic system, which made these devices attractive across studies for optimization of immunotherapy. Here, we reviewed the application of microfluidic systems in modeling TME and the potential of these devices in predicting and monitoring immunotherapy effects.

## Introduction

The tumor microenvironment (TME), as the soil of tumor growth and metastasis, is a complex and dynamic ecosystem mainly consisting of cellular components (i.e., stromal cells, immune cells, tumor cells) and noncellular components (i.e., extracellular matrix (ECM), vascular networks, cytokines, chemokines, etc.). The stromal cells including but not limited to endothelial cells, mesenchymal cells, and fibroblasts are crucial in facilitating and sustaining tumor cells. Within the TME, tumor cells mostly communicated with other cells through ECM and the secretion of molecules such as cytokines, growth factors, and lipid mediators (Hanahan and Coussens, 2012). The various immune cells play an important role in the complexity of TME (Hinshaw and Shevde, 2019). On the one hand, immune cells could be tumor-suppressive to inhibit tumor progressions, like the ability of CD8 T cells and natural killer (NK) cells to directly kill tumor cells. On the other hand, the immune cells could be tumor-supporting, such as myeloid-derived suppressor cells (MDSCs), regulatory T cells (Tregs), and type 2-polarized macrophages (M2), promoted the proliferation, metastatic dissemination even immune evasion of tumors (Hinshaw and Shevde, 2019).

The complex and vital role of TME in oncogenesis and tumor progression promoted a significantly increased number of relevant research, in which the multi-omics analysis showed promising advantages in recognizing the complexity of TME and tumor immunological heterogeneity. The multi-omics data analysis was also adopted to investigate the correlation between the genetic or epigenetic characteristics and the TME infiltration pattern in lung adenocarcinoma (LUAD) based on The Cancer Genome Atlas (TCGA) database, and successfully constructed a significant prognostic model (Zhang et al., 2020). Additionally, the TME of urothelial cancer (UC) has been comprehensively evaluated by the use of multi-omics analysis (Chu et al., 2021). A computational tool was developed by Zeng et al. (2021) for effective Immuno-Oncology Biological Research (IOBR). This tool not only succeed in decoding the TME and signature but also promoted the exploration of the immune-tumor interactions based on multi-omics analysis.

With the increasing evidence revealing the significance of TME in regulating tumor progression and the response to anti-tumor therapy, immunotherapies targeting TME have been widely expanded these years (Pitt et al., 2016a; Bejarano et al., 2021). The development of immune checkpoint blockade (ICB) immunotherapy started a new era of tumor therapy. The immune checkpoint inhibitors (ICIs) targeted cytotoxic T-lymphocyte-associated protein 4 (CTLA4) or the programmed cell death protein 1/programmed cell death ligand 1 (PD-1/PD-L1) axis have been applicated in clinical treatment and at a certain degree improved prognosis of patients with malignant tumors including melanoma, non–small cell lung cancer (NSCLC), head and neck cancer, renal cell cancer, urothelial carcinoma (Brahmer et al., 2012; Powles et al., 2014; Sharma and Allison, 2015), etc. In addition, many other therapeutic drugs targeting novel immune checkpoints (i.e., TIGIT, LAG3, TIM3) are being tested in clinical trials (Bejarano et al., 2021).

Nonetheless, the effect of immunotherapy is still unsatisfactory as the increased resistance and the comparatively low response of patients to these treatments (Pitt et al., 2016b; Ventola, 2017; Yarchoan et al., 2017). Thus, a huge number of researches focused on exploring effectively predictive biomarkers or combination therapies to improve clinical outcomes of immunotherapy. In this regard, microfluidic-based devices enabled mimicking the whole TME *in vitro*, have been widely used in modeling the TME of different tumors. Therefore, microfluidic technology promoted research on optimizing tumor immunotherapy furtherly based on the various microfluidic tumor models.

## Microfluidic technology

Microfluidics is a rapidly developed technology that made it possible to manipulate fluids flowing in channels of tens to hundreds of micrometers in size (Whitesides, 2006). With the consistent improvement of past years, microfluidics has exhibited excellent properties and has been applicated in diverse files including chemistry, engineering, biology, as well as medicine (Sackmann et al., 2014).

The manufacturing of microfluidic devices generally begins with using photolithography to create the mold, and then PDMS or other alternative materials were poured into the mold and cured, finally to form a PDMS microfluidic device with hollow microchannels. The unique microfluidic channels enabled the precise manipulation of flow such as mixture and separation of tiny fluids, chemical reactions, and microanalysis, which made the microfluidic chips attractive in the screening of rare cells, gene sequencing, separation and analysis of single-cell, extraction and purification of information RNA and so on. In this regard, microfluidic technology exhibited great potential in single-cell-omics analyses. For instance, an integrated proteomics chip (iProChip) based on microfluidic technology was designed and coupled with data-independent acquisition (DIA) mass spectrometry (MS) for the in-depth microproteomics identification and quantification (Gebreyesus et al., 2022). This microfluidic chip demonstrated sensitivity and robustness in the analyses of on average ∼1,500 protein groups across 20 single cells. In addition, the traditional single-cell genetic studies lack the spatial information of the cell, while the deterministic barcoding in tissue for spatial omics sequencing (DBiT-seq) could be a promising solution to this problem (Liu et al., 2020). The DBiT-seq is based on the principle of encoding tissues on chips using microfluidic technology, and then using deterministic barcodes in the tissues for spatial multi-omics sequencing, thus enabling the co-mapping of mRNAs and proteins in tissue slides (Liu et al., 2020).

Furthermore, certain properties of microfluidic chips, also called labs-on-chips, such as light size, low sample dose, accurate control of fluids, rapid, and parallel sample processing, have prompted the increasing application of organs-on-chips or tissues-on-chips in tumor-relevant research. Microfluidic chips, made of optical plastic, glass, PDMS, or other special polymers, are microfluidic devices designed for cell culture. Different from traditional 2D cell culture models, organ chips with microchannels allowed the fluids to flow across the cell chambers, which enabled the recapitulation for *in vivo* physical conditions such as vascular perfusion, air-liquid interfaces, shear stresses as well as the physical and chemical gradients (Murugesan et al., 2017; Liu et al., 2021). For instance, microfluidic chips were used to culture the human‐induced pluripotent stem cells (hiPSCs)‐derived hepatocytes-like cells (HLCs) (Danoy et al., 2021). And the multi‐omics analysis of the chip demonstrated a typical signature of a liver regenerative process, which provided an original overview of the sophisticated mechanisms of liver regeneration by the use of microfluidic technology (Danoy et al., 2021). In addition, some sophisticated organ chips even succeed in modeling organ-relevant mechanical activity by manipulating the organotypic tissue interfaces with the designed hollow side chambers (Choi et al., 2015; Hassell et al., 2017). Microfluidic chips have shown prominent advantages in faithfully and precisely recapitulating the physiology and pathophysiology at the organ-level and tissue-level in a series of studies on gut (Beaurivage et al., 2019; Xiang et al., 2020), lung (Huh et al., 2010; Benam et al., 2016; Zamprogno et al., 2021), kidney tubules and glomeruli, bone marrow, and so on (Jang et al., 2013; Torisawa et al., 2014; Musah et al., 2018).

## Microfluidics in modeling tumor microenvironment

Given the unique properties of the microfluidic system in recapitulating the structure, function, physiological and pathological characteristics of human tissues and organs, these devices have been widely used to effectively mimic and analyze tumor microenvironment *in vitro*, and to compensate for the lack of complexity and heterogeneity of tumor microenvironment in traditional 2D cell culture. With the increased application of microfluidics in TME modeling, the successes of microdevices in replicating several steps of metastatic cascade (i.e., cancer cells invasion, migration and adhesion, intravasation and extravasation) revealed the great potential of microfluidic technology in cancer metastatic research.

**FIGURE 1:**
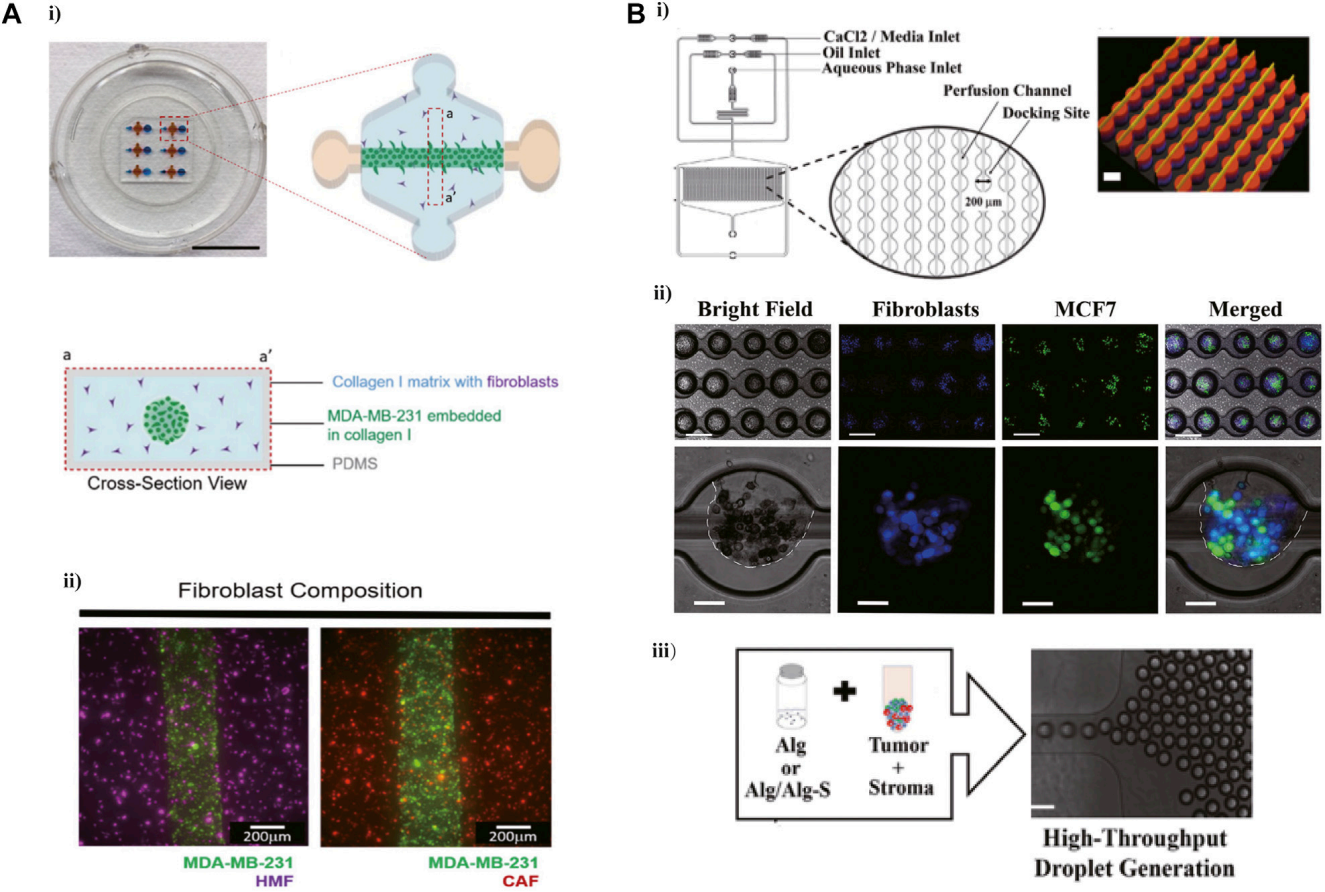
Microfluidic devices in ECM modeling. **(A)** A 3D microfluidic cell co-culture model was used to mimic the breast cancer TME and furtherly explore the effect of interactions between ECM and fibroblast on cancer invasion. i) The photograph and schematic depict of this microdevice. ii) A top view image showing the MDA-MB-231 cells co-cultured with NF or CAF 1 h after seeding. Reproduced from Karina et al. (Lugo-Cintron et al., 2020a) Copyright 2020 Cancers. **(B)** A high-throughput microfluidic system designed for recapitulating the breast cancer TME. i) Schematic representation of the microfluidic device. ii) Droplet generation, with MCF7 cells labeled with CFSE (green) and CCD1 129SK human mammary fibroblasts labeled with CMAC (blue). iii) Mixed the cells in Alg or Alg/Alg-S hydrogels to generate scaffolds, and then the mixture was infused into the device for droplet generation and final cross-linking. Reproduced from ref. (Berger Fridman et al., 2021) with permission from Acta Biomaterialia.

An increased number of studies are focused on the application of microfluidic technology in modeling the extracellular matrix (ECM) of TME which could promote the invasion, migration, and metastasis of cancer. For example, a microfluidic device created via the microfluidic called LumeNEXT was adopted to mimic the breast cancer TME *in vitro*, and furtherly explored the effect of interactions between ECM and fibroblast on cancer invasion (Figure 1A) (Lugo-Cintron et al., 2020a). This device contained several tumor lumens filled with the mixed solution of breast cancer cells and collagen and surrounded by the collagen matrix containing fibroblasts, aimed to simulate the interaction between cancer cells and stromal cells. It was found that the migration of MDA-MB-231 cells was significantly increased when co-cultured with matrix-embedded cancer-associated fibroblasts (CAFs) compared with those seeded in the normal fibroblasts matrix (HMFs) in this microfluidic device. In another 3D cell culturing microfluidic device, the solution mixed hydrogel and SUM-159 breast cancer cell obtained from a TNBC patient was injected into the tumor region, and the type I collagen contained the CAF or normal fibroblasts (NF) was added to the stromal region, modeling the cancer cells migrate into the normal region and detecting the influence of CAF on this invasion (Truong et al., 2019). It was found that compared with NFs, CAF expressed a tumor-promoting behavior showed as enhancement of cancer cells proliferation, migration as well as cell aspect ratio. Meanwhile, this research showed that CAF improves the invasion of breast cancer cells *via* inducing the expression of glycoprotein non-metastatic B (GPNMB) on cancer cells in the 3D microenvironment. Another 3D culture model, integrated 3D tumor spheroids (TSs), and CAF on a microfluidic chip was used to recapitulate the interaction between TSs and fibroblasts (Jeong et al., 2016). The alginate (Alg) or alginate-alginate sulfate (Alg/Alg-S) hydrogels were applied to generate the tumor-stoma scaffolds along with breast cancer cells in a high throughput microfluidic system, recapitulating the breast cancer TME Figure 1B (Berger Fridman et al., 2021). This study demonstrated a transformation of macrophages from proinflammatory to immunosuppressive phenotype in Alg/Alg-S hydrogel and confirmed the proteins involved in immunomodulation and cellular interactions upregulating within Alg/Alg-S. This high throughput microfluidic device contained 1,000 docking sites ranking as 40 rows and 25 columns, supporting a rapid and efficient way to generate a complex and dynamic breast TME *in vitro* model.

**FIGURE 2:**
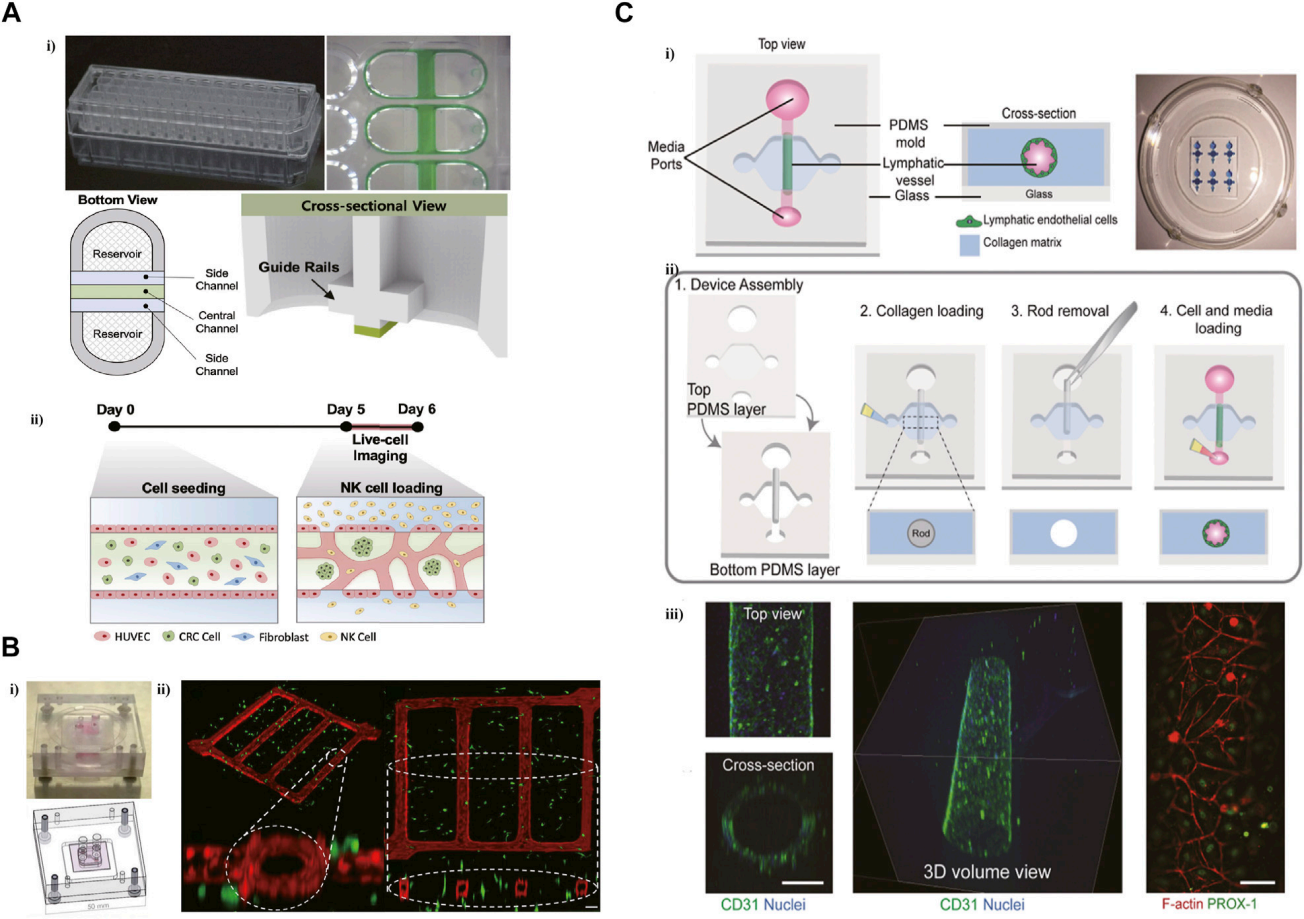
Microfluidic devices in recapitulating the tumor vasculature and lymphatic vessel. **(A)** A high-throughput microfluidic platform with three parallel microchannels designed for modeling the tumor vasculature. i) The photograph and schematic depict of this microdevice. ii) The blood vessel networks were formed with endothelial cells, fibroblasts, and CRC cells within the central channel. Reproduced from Song et al. (Song et al., 2021) Copyright 2021 Song, Choi, Koh, Park, Yu, Kang, Kim, Cho and Jeon. **(B)** A network platform with interconnected microfluidic channels for modeling a highly vascularized system. i) The design of this microvascular network platform. ii) Isometric view of the co-culture network. Reproduced from ref. (Michna et al., 2018) with permission from Biotechnol Bioeng. **(C)** A microfluidic device was designed to generate lymphatic vessels (LVs) within a collagen hydrogel. i) Schematic representation of the microfluidic device. ii) Microdevice design and fabrication scheme. iii) Confocal image of the lymphatic vessel with 3D tubular structure. Reproduced from ref. (Lugo-Cintron et al., 2020b) with permission from The Royal Society of Chemistry.

Microfluidic technology promoted the development of a complicated vascularized *in vitro* model to imitate the TME. In a microfluidic platform with three parallel microchannels, modeling the tumor vasculature through the generation of blood vessel networks formed with endothelial cells, fibroblasts, and colorectal cancer (CRC) cells within the central channel (Figure 2A) (Song et al., 2021). And then introduced NK cells into the vessel *via* the side channel. It was found that NK cells presented high cytotoxicity in consensus molecular subtypes1 (CMS1) CRC cells in this tumor vasculature model. This platform contained 28 wells that allowed performing various high-throughput experiments ranging from interactions of immune and cancer cells within TME to drug screening for immunotherapy. A network platform with interconnected microfluidic channels was created to mimic a highly vascularized system. This novel microfluidic platform imitated the interactions between tumor cells and vasculature, and succeed in modeling vessel leakiness presented in the TME (Figure 2B) (Michna et al., 2018).

Lymphatic endothelial cells (LECs), lacking basement membrane, are leakier than blood vessels, promoting the metastasis of cancer cells. To decipher the influence of ECM on the lymphatic vessel (LV) in Bca, a microfluidic device was designed to generate LVs within a collagen hydrogel (Figure 2C) (Lugo-Cintron et al., 2020b). This microfluidic system elucidated the change of LV toward activated phenotype via the increased secretion of IL-6 induced by a dense ECM. The secretion of IL-6 can also increase the leakiness of LV in this microfluidic model.

**FIGURE 3:**
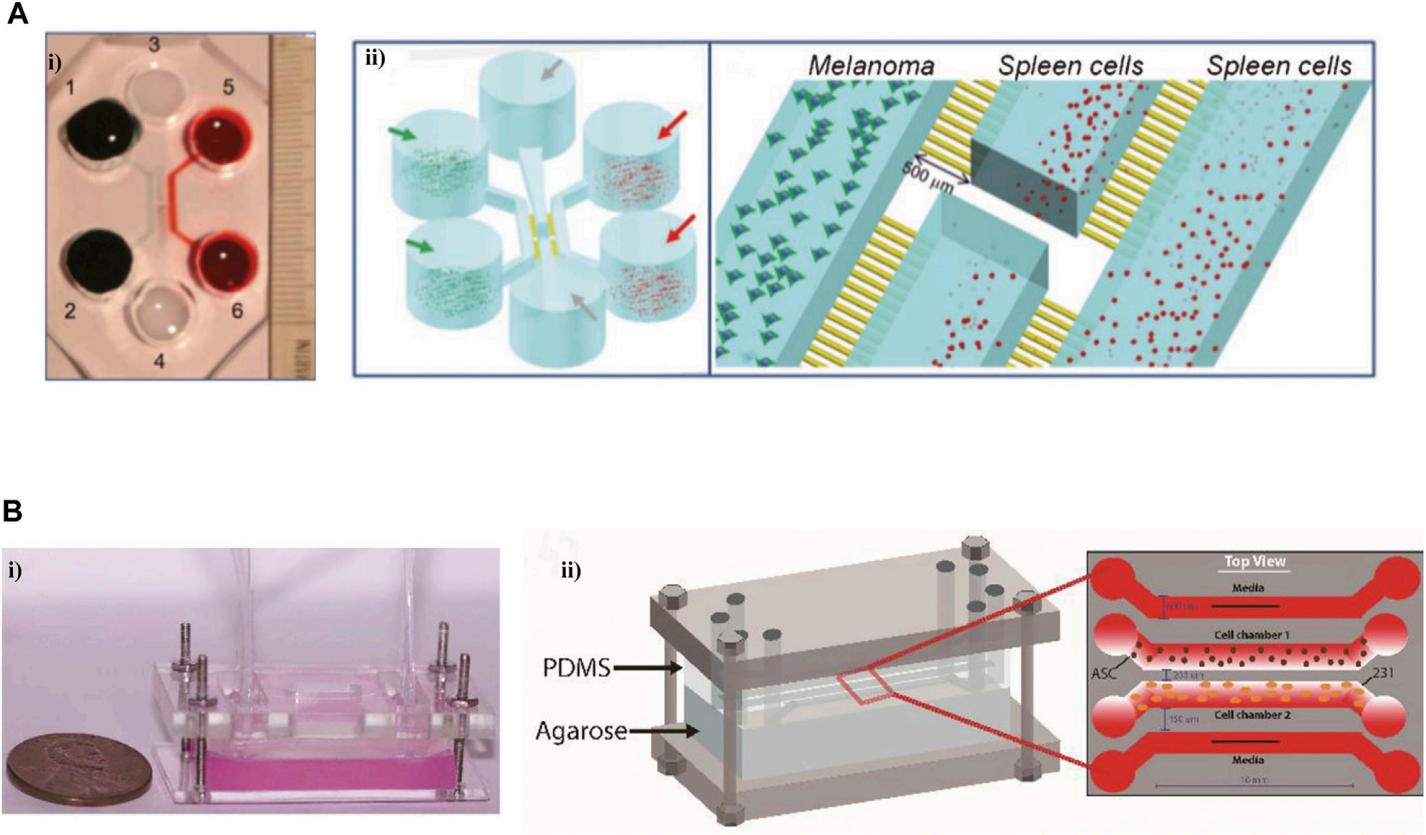
Microfluidic devices in modeling the interactions between immune cells and tumor cells. **(A)** An on-chip model to investigate the interactions between cancer and the immune system. i) The photograph of this microfluidic chip. ii) The schematic views of this platform. Reproduced from ref. (Businaro et al., 2013) with permission from The Royal Society of Chemistry. **(B)** A novel microfluidic platform imitated the interactions between tumor cells and vasculature, and succeed in modeling vessel leakiness presented in the TME i) The image of this device. ii) The schematic of this microdevice. Reproduced from ref. (Rahman et al., 2020) with permission from The Royal Society of Chemistry.

Microfluidic models have also exhibited significant advantages in recapitulating the interplays between cancer and the immune system. A tumor-on-chip platform allowed the interplay of cellular and non-cellular components, modeling the TME and exploring the influence of TME on immune cell recruitment (Aung et al., 2020). It was shown that the presence of the hypoxic condition and NK cells improved T-cell recruitment in this tumor-on-chip model. The intercellular communication within TME is crucial for supporting the tumor phenotype. Using a “flow-free” microfluidic device with four channels to simulate the crosstalk between two cell types (Figure 3B) (Rahman et al., 2020). The MDA-MB-231 cells co-cultured with adipose-derived stem cells (ASCs) exhibited aggressive phenotype and polarization toward ASCs. Interferon regulatory factor 8 (IRF-8) is a necessary transcription factor for immune response induction. The B16 cells and immune cells obtained from WT and IRF-8 KO mice were co-cultured in a microfluidic chip (Figure 3A) (Businaro et al., 2013). In this on-chip model, WT spleen cells showed an increased migration toward B16 cells via microchannels, and a tighter interaction with cancer cells compared to IRF-8 KO spleen cells. B16 cells expressed a more aggressive phenotype when co-cultured with IRF-8 KO spleen cells, which was confirmed in another research (Mattei et al., 2014).

## The microfluidic in immunotherapy

**FIGURE 4:**
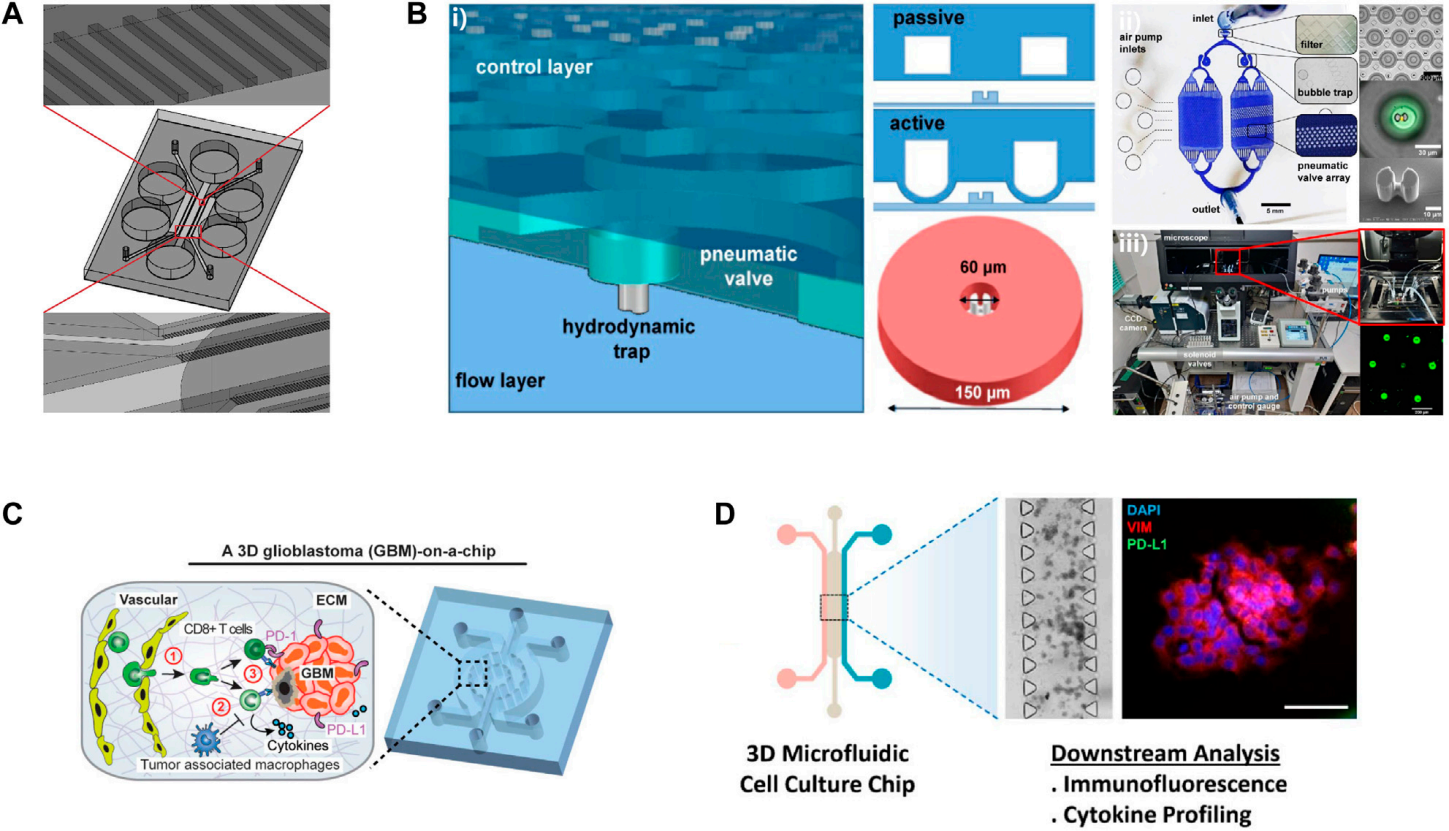
Examples of microfluidic devices for immunotherapy optimization. **(A)**The schematic of this microdevice. Reproduced from ref. (Al-Samadi et al., 2019) with permission from Elsevier Inc. **(B)** i) An illustration of this microfluidic device. ii) The microfluidic platform for single cell compartmentalization. iii) The experimental set-up of this study. Reproduced from ref. (Briones et al., 2020) Copyright Jonathan C.. Briones1, Wilfred V. Espulgar1, Shohei Koyama et al. **(C)** Reproduced from ref. (Cui et al., 2020) Copyright Cui et al. **(D)** Reproduced from ref. (Aboulkheyr Es et al., 2021) with permission from 2020 Wiley Periodicals LLC.

Aimed to improve the effects of tumor immunotherapy, more and more research has focused on testing the response of patients to ICB *in vitro* microfluidic systems. The probable effect of immunotherapy on head and neck squamous cell carcinomas (HNSCC) patients was determined using an *in vitro* 3D microfluidic chip, which loaded different ICIs, Indoleamine 2, 3-dioxygenase 1 (IDO1) inhibitors, and PD-L1 antibodies (Figure 4A) (Al-Samadi et al., 2019). It was observed that the IDO1 inhibitor induced the migration of immune cells toward both HSC-3 cells and cancer cells isolated from HNSCC patients in this microfluidic device. This study provided a new method to test the efficacy of ICIs for patients on a humanized microfluidic chip. To recapitulate the function of ICB *in vitro*, a 3D microfluidic device was adopted to culture organotypic tumor spheroids derived from murine (MDOTS) or patients (PDOTS) (Jenkins et al., 2018). The results of functional assays revealed the capability of MDOTS/PDOTS in modeling response to PD-1 blockade *in vitro*, which was confirmed in subsequent research (Aref et al., 2018). With the capability of identifying specific T cells necessary for effective tumor immunotherapy through measuring the activity of granzyme B, a microfluidic platform has shown the potential in evaluating the sensitivity of immunotherapy (Figure 4B) (Briones et al., 2020). To enhance the efficacy of ICB, it was attractive to explore the combination of immunotherapy with new therapy manners by the use of microfluidic systems. Researchers succeed in determining the role of MSCs in inducing PD-L1 expression via co-culturing the MCF cells and mesenchymal stem cells (MSCs) in a 3D microfluidic cell culture device (Figure 4D) (Aboulkheyr Es et al., 2021). The results of functional assays revealed that MSCs induced the expression of PD-L1 on breast cancer cells via the secretion of CCL5. This study supported a new alternative method for the combination of ICIs with pirfenidone (PFD) which was previously demonstrated could significantly lower the PD-L1 expression level of metastatic cancer cells. Microfluidic systems also promoted the development of screening of immunotherapy for individuals. “GBM-on-a-chip,” a patient-specific microfluidic system was used to optimize immunotherapy for glioblastoma (GBM) patients with different subtypes (Figure 4C) (Cui et al., 2020).

## Discussion

The critical role of TME in mediating tumor progression and affecting therapeutic outcomes is more and more apparent with the increasing evidence from plenty of research. From this, various therapies targeting the various components of the TME have been developed, and some therapeutic drugs have improved patient prognosis to some extent (Bejarano et al., 2021). Among these therapeutic drugs, ICIs targeted PD-1/PD-L1 and CTLA-4 have induced unprecedented responses in some patients with advanced cancers, nonetheless, the limited clinical response restricted the universal clinical application of ICB therapy across multiple cancers (Pitt et al., 2016b; Ventola, 2017; Yarchoan et al., 2017). To screen patients who could benefit from immunotherapy and optimize the treatment, many researchers are focused on determining specific prognostic indicators and exploring the combination of immunotherapy with other approaches using *in vitro* models. As the traditional 2D cell culture and murine model could not recapitulate the faithful TME of humans, microfluidic devices, which enabled mimicking the dynamic and complex TME, have become more and more attractive in creating the *in vitro* tumor models.

With the continuous progression over the past years, microfluidic devices, based on microfluidic technology, have been developed to recapitulate the physiological and pathological condition of humans *in vitro* (Hassell et al., 2017). Microfluidic devices have significant advantages in imitating not only vascular perfusion, air-liquid interfaces, shear stresses as well as the physical and chemical gradients of physical conditions, but also the mechanical activity within organs or tissues of humans (Choi et al., 2015; Murugesan et al., 2017; Liu et al., 2021). As for the imitation of pathological conditions, microfluidic devices are mainly applicated in modeling the TME of tumor progression. ECM, the crucial noncellular component of the TME, primarily composed of collagen, non-collagen, elastin, and proteoglycan, could promote tumor growth and progression through transmitting signals secreted from fibroblasts and epithelial cells within TME (Biteau et al., 2011). By the use of microfluidic devices to co-culture tumor cells with different matrices containing CAFs or HMFs, researchers succeed in modeling the interaction between ECM and stromal cells and determining the promotion of such interaction on tumor progression (Lugo-Cintron et al., 2020a). Furthermore, the blood and lymphatic vascular networks, the same important noncellular component of the TME, have been successfully recapitulated on the microfluidic platforms as well (Michna et al., 2018; Lugo-Cintron et al., 2020b; Song et al., 2021). The interactions between immune cells and cancer cells determined the response of anti-tumor therapy. Using microfluidic devices to mimic the interplay between tumor cells and immune cells *in vitro* (Businaro et al., 2013; Aung et al., 2020; Rahman et al., 2020), has been confirmed with more significant advantages compared with traditional murine models (Mattei et al., 2014). According to the distribution site, ECM can be divided into basement membrane and interstitial matrix, and the tumor metastasis start from the invasion of cancer cells toward the basement membrane and migrate to a remote site (Friedl and Alexander, 2011; Wolf et al., 2013). In this review, we introduced the studies on microfluidic devices in modeling ECM, cancer cells invasion, and the leakiness of LV, and the specific microfluidic models even presented EMT phenotypes. These findings supported the prominent significance of microfluidic systems in modeling TME and the metastatic TME. Given the significance of microfluidic devices in modeling the immune cells interplay with tumor cells within the TME, increased research has been reported to test the response of patients to ICIs and develop new approaches to combine with immunotherapy via microfluidic *in vitro* model (Table 1) (Aref et al., 2018; Jenkins et al., 2018; Al-Samadi et al., 2019; Briones et al., 2020; Cui et al., 2020; Aboulkheyr Es et al., 2021).

**TABLE 1:** Summary of reviewed literature.

Applications	Experiment design	Microfluidic device	Findings	Refs
Modeling ECM and the interaction between ECM and tumor cells	Co-culture cancer cells or 3D tumor spheroids with the matrix containing collagen and fibroblasts	LumeNEXT	Co-culturing with CAFs promoted the migration of MDA-MB-231 cells	Lugo-Cintron et al. (2020a)
		3D microfluidic co-culture system	CAFs enhanced breast cancer cells invasion and migration by inducing the expression of GPNMB	Truong et al. (2019)
		microfluidic chip integrated 3D tumor spheroid and CAFs	Co-culturing with CAFs promoted the migration of 3D tumor spheroids cells	Jeong et al. (2016)
	Generate a tumor-stoma scaffolds using Alg or Alg/Alg-S hydrogel	A high-throughput microfluidic system	Alg/Alg-S induced complex in vivo-like alteration including EMT phenotypes and transformation of M1 to M2	Berger Fridman et al. (2021)
Modeling tumor vasculature	Generate blood vessel networks	A high-throughput microfluidic platform with 3 parallel microchannels	NK cells presented high cytotoxicity in CMS1 CRC cells	Song et al. (2021)
		A network platform with interconnected microfluidic channels	This microfluidic platform imitated the interactions between tumor cells and vasculature, and succeed in modeling vessel leakiness presented in the TME.	Michna et al. (2018)
Modeling lymphatic vessel (LV)	Create the lumen structure with primary human lymphatic endothelial cells (HLECs) within collagen hydrogels	3D microfluidic co-culture system	The dense ECM promoted LV transformed toward activated phenotype via increasing secretion of IL-6	Lugo-Cintron et al. (2020b)
Modeling the interactions between immune cells and tumor cells	Examine the effect of cancer cell-monocyte interaction on T-cell recruitment	A tumor-on-a-chip platform	The presence of the hypoxic condition and NK cells improved T-cell recruitment in this tumor-on-chip model	Aung et al. (2020)
	Co-culture triple-negative MDA-MB-231 breast cancer cells and ASCs	A “flow-free” microfluidic device with 4 channels	ASCs promoted the aggressive phenotype and polarization toward ASCs of MDA-MB-231 cells	Rahman et al. (2020)
	B16 cells were co-cultured with immune cells obtained from WT and IRF-8 KO mice	A microfluidic chip	WT spleen cells showed an increased migration toward B16 cells; B16 cells expressed a more aggressive phenotype when co-cultured with IRF-8 KO spleen cells	Businaro et al. (2013); Mattei et al. (2014)
Testing the efficacy of immunotherapy	Evaluate the migration of immune cells towards cancer cells and the cancer cell proliferation rate	3D microfluidic chip loaded with different immune checkpoint inhibitors, PD-L1 antibody, and IDO 1 inhibitor	IDO1 inhibitor induced the migration of immune cells toward both HSC-3 cells and cancer cells isolated from HNSCC patients in this microfluidic device	Al-Samadi et al. (2019)
	Measure the ability to interrogate *ex vivo* response to ICB using MDOTS/PDOTS	A 3D microfluidic culture system	This microfluidic device succeeded in modeling response to PD-1 blockade *in vitro*	Aref et al. (2018); Jenkins et al. (2018)
	Measure the activity of granzyme B to identify specific T cells necessary for effective tumor immunotherapy	A microfluidic platform	This microfluidic platform has shown the potential in evaluating the sensitivity of immunotherapy by measuring the activity of granzyme B	Briones et al. (2020)
Exploring the combination of immunotherapy with new therapy manners	Co-culture the MCF cells and MSCs	A 3D microfluidic cell culture chip	Supported a new alternative method for the combination of ICIs with PFD	Aboulkheyr Es et al. (2021)
	Identify potential therapy responses in a cohort of molecularly distinct GBM patients	A GBM-on-a-Chip system	This microfluidic chip enabled the personalized screening of immunotherapies for GBM patients	Cui et al. (2020)

In contrast with conventional methods, microfluidics provides a more rapid and cost-effective technic to construct more controllable and reproducible methods for drug delivery and screening (Bjornmalm et al., 2014; Damiati et al., 2018). The drug carries synthesized by microfluidic devices, such as microcapsules, nanoemulsions, and nanoparticles can be efficiently delivered to the target regions at expected speed and time therefore to improve the drug efficacy. Microfluidic systems enable the multiplexed drug screening in a simple and high-throughput manner, from the cell level to the organ-, even whole-body levels. Recent studies also revealed the impressive progresses of microfluidic systems in modeling the drug resistance tumor models, which would promote the development of multidrug delivery treatment strategies (Rahmanian et al., 2021).

Although microfluidic devices have exhibited comparable advantages over traditional 2D culture systems for modeling the TME in preclinical research, there are some challenges to further application for these devices. For instance, PDMS, the primary material of microfluidic devices, has been demonstrated to absorb small molecules which could affect the drug screening studies outcomes (Toepke and Beebe, 2006; Regehr et al., 2009). While alternative materials such as polystyrene, cyclic olefin copolymer, and paper have been explored to mitigate this problem, the requirement of re-thinking of component design for different materials remained this problem intractable (Chin et al., 2011; von Lode, 2005; Browne et al., 2009). Additionally, the variable parameters of culture conditions in 3D microfluidic culture systems, including medium, components, and concentration of ECM and molecules like cytokines, chemokines, and growth factors could influence the function of these systems. Moreover, expanded research to perform the comprehensive evaluation and analyses of 3D tumor models based on microfluidics is required for further application in immunotherapy improvement. Several limitations of microfluidic devices also limited their application as the tool to predict the effect of patients on immunotherapies. Firstly, mass production is the obstruction of microfluidic devices for clinical application. On the one hand, mass production needs the manufacturability and durability of a series of microfluidic devices, which are limited by PDMS. On the other hand, the manipulation and analysis for studies performed on the microfluidic platforms are heavily dependent on necessary external equipment and high-resolution imaging as well as the time-consuming image analysis, which is considered to lack convenience and applicability for clinical application. It should be noted that the majority of studies on microfluidic devices are proof-of-concept research, thus it was necessary to carry out more clinical trials testing the utility of these devices. The multidisciplinary collaborative work on producing more convenient and applicable microfluidic devices is indispensable to achieve this possibility.

Microfluidic devices possess the huge potential to serve as a predictive and effective tool for immunotherapies to optimize the treatment of tumor patients. As CTCs and exosomes isolated using the microfluidic-based device have shown specific significance in the prediction and monitoring of response to ICIs, it is expected to produce an integrated microfluidic-based device that allowed high-throughput and automated assays for isolation and diagnostic test for patients. In the future, the breakthrough of microfluidic technology may simplify the design and manufacture of microfluidic systems, enable the minimization of these devices, and promote their application in clinical diagnosis and treatment.

## Conclusion

In summary, we briefly introduced the microfluidics including the application in multi-omics analyses, and then focused on the microfluidic technology applicated in the TME modeling, finally reviewed the potential of microfluidic systems for further application in immunotherapies according to their capacities in recapitulating the TME *in vitro*. We also discussed the challenges and future of microfluidic devices in clinical application. Overall, we are looking forward to microfluidic systems that can 1 day realize their significance in clinical practice and promote cancer immunotherapies and precision medicine.
